# Detection of postural sway abnormalities by wireless inertial sensors in minimally disabled patients with multiple sclerosis: a case–control study

**DOI:** 10.1186/s12984-015-0066-9

**Published:** 2015-09-01

**Authors:** Andrew J. Solomon, Jesse V. Jacobs, Karen V. Lomond, Sharon M. Henry

**Affiliations:** Department of Neurological Sciences, University of Vermont College of Medicine, University Health Center - Arnold 2, 1 South Prospect Street, Burlington, VT 05401 USA; Department of Rehabilitation and Movement Science, University of Vermont, Burlington, VT USA; School of Health Sciences, Central Michigan University, Mt Pleasant, MI USA

## Abstract

**Background:**

Common clinical neurological exams can be insensitive to balance and mobility impairment at the early stages of multiple sclerosis (MS) and may not correspond with patient reports. Instrumented measurement of standing postural sway with inertial motion sensors may provide sensitive measures of balance impairment and better correspond with patient reports.

**Methods:**

While wearing wireless inertial sensors, 20 subjects with MS – Expanded Disability Status Scale of less than 3.0 and a Timed 25 Foot Walk of 5 sec or less – and 20 age- and sex-matched control subjects stood with eyes open and eyes closed on a foam surface. Forty-six outcome measures of postural sway were derived. A stepwise logistic regression model determined which measures of instrumented sway provide independent predictors of group status. Subjects with MS also completed the Activities-Specific Balance Confidence (ABC) scale and the 12-Item MS Walking Scale (MSWS-12) as measures of subject-reported balance and mobility impairment.

**Results:**

The regression model identified medio-lateral sway path length and medio-lateral range of sway acceleration amplitude, each in the eyes-open condition, as the only two significant independent predictors to differentiate subjects with MS from those without MS (model chi-squared = 34.55, *p* < 0.0001): accuracy = 87.5 %, positive likelihood ratio = 6 (2.09–17.21), negative likelihood ratio = 0.12 (0.03–0.44). Range of sway acceleration amplitude significantly correlated with both ABC (Spearman’s *r* = −0.567, *p* = 0.009) and MSWS-12 scores (Spearman’s *r* = −0.590, *p* = 0.006).

**Conclusions:**

Postural sway abnormalities in subjects with MS who are minimally disabled were detected using wireless inertial sensors and may signify a superior sensitivity to identify balance impairment prior to developing clinically evident disability or impaired gait speed. Further study is needed to confirm the clinical significance and predictive value of these objectively identified balance impairments.

## Background

Balance impairment is common in people with multiple sclerosis (MS) and frequently impacts quality of life by decreasing mobility and increasing the risk for falls [1, 2]. The causes of balance dysfunction in MS are not well understood. More sensitive measures of balance impairment are needed to better understand mechanisms of postural control affected by MS.

A number of clinical and self-reported measures have been utilized to evaluate balance impairment in patients with MS. Current clinical measures of walking and balance in MS frequently used for clinical trials and clinical decision-making include the Expanded Disability Status Scale (EDSS) and Timed 25-Foot Walk (T25FW) Test. The EDSS, however, is heavily weighted toward ambulatory disability and is less sensitive to other dimensions of disability, may not perform well in patients with milder disability, and has been critiqued for insensitivity to detect changes in level of disability [3–5]. A worsening of 20 % or more on the T25FW appears to reliably indicate progression of disability, however patients minimally affected by MS frequently walk 25 feet between 3 and 5 sec, [6, 7] which is below the 6-second threshold associated with accrual of significant disability [8]. Thus, a 20 % change in walk time may be relatively insensitive to disability progression during the early stages of MS. The Brief-BESTest [9] is an 8-item clinical assessment of balance impairment that was specifically designed to evaluate multiple contexts of balance and gait impairment. The Brief-BESTest has shown promise to identify fall risk in people with MS and is sensitive to change following a balance exercise intervention for people with MS [9, 10]. The Brief-BESTest, therefore, may offer a more comprehensive clinical evaluation of balance impairment [11] than the EDSS or T25FW, but it remains unclear if this clinical tool better differentiates people at early stages of MS from people without MS, or if instrumented measures of balance impairment might be necessary.

In addition to clinical assessments often being insensitive to differences between people with early-stage MS and people without MS, patients with MS sometimes report difficulties with mobility or balance despite normal clinical tests [12]. The MS Walking Scale-12 (MSWS-12) [13, 14] is a widely used self-reported rating scale used to assess the impact of MS on walking ability that includes questions concerning walking and balance impairment. The Activities-Specific Balance Confidence (ABC) Scale is a 16-item self-report measure in which patients rate their balance confidence for performing activities [15] and has demonstrated validity for patients with MS, [16] as well as associations with objective, instrumented measures of balance [1]. An effective balance assessment, therefore, should not only be sensitive to early changes in balance and mobility due to MS, but it should also better reflect patient reports of balance and mobility impairment as identified by measures such as the ABC scale and MSWS-12.

There remains a significant need for an objective, practical, sensitive and predictive clinical measure of balance impairment in MS that shows a strong association with patient-reported symptoms. Use of body-worn wireless inertial sensors may provide more sensitive measures of balance impairment than current clinical measures while also enabling ease of clinical implementation compared to traditional kinematic methodology [17]. Measurements using inertial sensors have demonstrated in patients with other neurological disease, such as Parkinson’s disease, an ability to discriminate subjects with early disease compared to controls, correlation with disease severity, and sensitivity to early progression of postural sway abnormalities [18–21]. In a study by Spain and colleagues, such sensors have demonstrated abnormal standing postural sway in subjects with MS who present with normal walking times when compared to subjects without MS [22]. The predictive capacity of these measures to differentiate groups with and without MS, however, was of only moderate strength (areas under the receiver operating characteristic curves ranged from 0.39 to 0.72) [22]. In that study by Spain and colleagues, though, subjects were only tested for standing postural sway on a firm surface; other studies have suggested that postural sway is most impacted by MS when modifying sensory input, such as when standing on a compliant surface [23]. Thus, it remains necessary to (1) maximize the potential sensitivity of body-worn sensors to differentiate subjects with and without MS by testing conditions known to do so, and (2) examine the predictive capacity of instrumented standing sway measures to differentiate subjects without MS from subjects selected specifically to have early-stage MS.

The purpose of this study is to determine if instrumented measures of standing postural sway (using body-worn inertial motion sensors) can differentiate people with MS chosen for minimal impairment and clinically normal gait times from people without MS while also corresponding with subject-reported balance and gait difficulty. In spite of clinical tests suggesting minimal disability, such patients may identify problems with balance and mobility and, therefore, we included subject-reported measures to determine in this cohort with minimal impairments if instrumented measures correspond to subject perception of balance (ABC) and gait (MSWS-12). Prior studies have supported that a variety of instrumented measures can correspond to subject-reported measures of balance impairment in subjects with MS [1, 24, 25]. We thus hypothesized that instrumented measures of standing postural sway would differentiate subjects with and without MS, despite clinically normal gait speed and minimal clinically defined disability, and associate with self-reported measures of balance impairment. Unlike prior studies that have utilized body-worn inertial sensors [22, 26], this study evaluated stance on a compliant foam surface in order to determine if instrumented measures of sway in this more challenging condition can provide discriminative validity to identify people with minimally impaired MS from control subjects without MS.

An improved understanding of balance impairment in people with minimally disabling MS through more sensitive measures, such as those derived from body-worn inertial sensors, may allow the identification of biomarkers to guide early therapeutic interventions. Therapeutic decisions made before the onset of significant disability may play an important role in modifying disease trajectory and preserving mobility as well as quality of life.

## Methods

### Subjects

After written informed consent, 20 people with MS and 20 without MS completed the protocol, which was approved by the local Institutional Review Board. Inclusion criteria for the subjects with MS included: (1) diagnosis of MS with an EDSS of less than 3.0, (2) T25FW of 5 sec or less, (3) age 18–70, (4) ability to stand unassisted, and (5) an ability to walk without an assistive device, including a cane or walker. Exclusion criteria for the subjects with MS included: (1) current use of Dalfampridine medication, (2) use of an ankle-foot orthotic, (3) MS relapse in the last 3 months, (4) previous foot, knee, or hip surgery, (5) severe pain that limited mobility, (6) significant vision impairment limiting mobility or balance, and (7) any additional neurological or medical conditions that may impair walking or balance, including but not limited to stroke, neuropathy, low back pain, depression, or arthritis.

The control group of subjects without MS included subjects aged 18–70, and exclusion criteria included any known medical diagnoses or current medication that might impair or influence balance. These included, but were not limited to: (1) any diagnosed neurological disorder or neurological symptoms other than episodic migraine, (2) any diagnosed inner ear or vestibular disorder, (3) persistent musculoskeletal pain, (4) a history of orthopedic surgery that might impair balance, including foot, ankle, leg, knee, hip, or spine surgery, (5) a current diagnosed psychiatric disorder such as depression or anxiety, or (6) use of medications that may affect the central nervous system, including opioids and benzodiazepines.

### Protocol

Prior to instrumented measurement of balance, only the subjects with MS completed the 12-Item MS Walking Scale (MSWS-12) and Activities-Specific Balance Confidence Scale (ABC).

Subjects with MS and control subjects without MS participated in the instrumented measurement of postural sway. The instrumented task was to stand on a foam surface with either eyes open or eyes closed. Body-worn sensors using the Opal system (©APDM, www.apdm.com) were utilized for the study. The Opal system consists of small, lightweight, body-worn sensors that contain 3-dimensional gyroscopes, accelerometers, and magnetometers. Six sensors were strapped to the subjects’ bodies at the sternum, lumbar area, wrists and ankles. The sensors wirelessly transmit their raw data at 120 Hz to a laptop data collection system.

Instrumented postural sway (iSWAY) was performed while subjects were standing on 10.16 cm of medium-density foam with their feet in a standardized position using a footplate. The footplate was 15.24 cm wide between the top of the feet and 10.48 cm between the heels, creating an angle of 17.3° between feet. The subjects were instructed to stand as still as possible for 30 sec with their arms crossed and hands on their shoulders. Three trials each were performed in an eyes-open (EO) and eyes-closed (EC) condition for a total of six trials. The subjects stepped off the foam between each trial for approximately 1 min to rest. The total time required to complete iSWAY for each individual was approximately 10 to 15 min, which included instructions for the task as well as placement and removal of sensors and software validation of each trial.

Following the instrumented measures, only the subjects with MS completed the T25FW, Brief-BESTest, [9] and an EDSS exam performed by a neurologist with post-doctoral subspecialty training in MS and certification to perform EDSS exams for clinical trials. Subjects with MS were offered time to rest as needed between each measure, no subject complained of fatigue after completion of the protocol.

### Data processing & analysis

Forty-six outcome measures were automatically derived and exported using the Mobility Lab software algorithms (©APDM) [17, 27]. These include multiple measures representing amount, velocity, acceleration, jerk, and spectral power of sway in the anterior-posterior and medial-lateral directions as well as in the combined horizontal plane. Since all 46 measures are readily available to a clinician or researcher, each was considered in the analysis following correction for multiple comparisons. Each subject’s measures were averaged by trial and visual condition for analysis. Clinical balance and gait impairments were tested by the Brief-BESTest [9] and T25FW test [8]. Sum Brief-BESTest scores and 25-foot walk times were derived for analysis. Subject-reported measures of balance and gait difficulty were derived from the average score of the ABC scale [15] and the percentage score of the MSWS-12 [13].

Because Shapiro-Wilks tests of normality often determined that the data did not meet requirements for normality, differences in each measure of instrumented sway between the groups with and without MS were determined using Mann–Whitney U tests. Because 46 measures of sway were determined and assessed twice (once for each visual condition; total = 92 comparisons), significance was set to a Bonferroni-corrected value of 0.00054. A forward, likelihood-ratio, stepwise logistic regression model was used to determine which measures of instrumented sway provide independent predictors of group status (i.e., subjects with early-stage MS versus subjects without MS). Variables entered into the model included only the measures that were significantly different between groups based on the Mann–Whitney U tests.

Spearman’s rho correlation coefficients were used to evaluate associations between the instrumented sway measures identified as significant independent predictors of group status and the subject-reported measures of balance and gait difficulty (the ABC scale and MSWS-12). Likewise, Spearman’s coefficients were used to evaluate associations between clinical exam scores (Brief-BESTest, T25FW, and EDSS) and the subject-reported measures of balance and gait difficulty. The Mann–Whitney U tests and regression analysis, thus, serve to test the hypothesis that instrumented measures of standing postural sway can differentiate subjects with early-stage MS from subjects without MS; the correlation analysis determined whether the instrumented measures of postural sway that differentiate subjects based on group status or the clinical-exam measures correspond with subject perceptions of balance and gait difficulty.

## Results

### Subject characteristics

Both groups with and without MS were comprised of four males and 16 females with mean (95 % CI) ages of 40 (35–45) years. Demographic and clinical characteristics of the group with MS are displayed in Table 1.

**Table 1:** MS cohort demographic and clinical characteristics

^a^Gender	Four male 16 female
^a^Age (95 % CI)	40 (35–45)
Years since diagnosis (95 % CI)	4 (1–7)
EDSS median (range)	2.0 (1.0–2.5)
T25FW (95 % CI)	3.9 (3.6–4.1)
Brief-BESTest (95 % CI)	22 (21–23)
ABC (95 % CI)	96 (93–99)
MSWS-12 (95 % CI)	14 (5–22)%

^a^Control subjects without MS were age and gender matched to subjects with MS

### Group differences in instrumented sway variables

In the eyes-open condition, following Bonferroni correction, 15 of the 46 instrumented measures of sway were significantly different between the groups with and without MS (Table 2); in the eyes-closed condition, 22 of the 46 measures were significantly different between groups (Table 3).

**Table 2:** Mean (95 % CI) measures of sway with significant group differences: eyes open

*Instrumented measure of sway	Group with MS	Control group	Mann–Whitney U (*p*-value)
ML jerk (m^2^/s^5^)	0.0092 (0.0064–0.0120)	0.0030 (0.0023–0.0037)	25 (*p* < 0.0001)
Horizontal sway area (m^2^/s^4^)	0.0086 (0.0064–0.0108)	0.0038 (0.0032–0.0045)	37 (*p* < 0.0001)
95 % confidence circle area (m^2^/s^4^)	0.099 (0.076–0.121)	0.049 (0.037–0.061)	63 (*p* = 0.0001)
95 % confidence ellipse area (m^2^/s^4^)	0.083 (0.060–0.105)	0.034 (0.028–0.041)	49 (*p* < 0.0001)
AP root mean square (m/s^2^)	0.084 (0.075–0.093)	0.062 (0.054–0.070)	73 (*p* = 0.00036)
Horizontal root mean square (m/s^2^)	0.101 (0.089–0.113)	0.071 (0.063–0.078)	62 (*p* < 0.0001)
ML root mean square (m/s^2^)	0.052 (0.042–0.062)	0.031 (0.027–0.036)	70 (*p* = 0.00026)
AP mean sway velocity (m/s)	0.221 (0.189–0.254)	0.149 (0.116–0.183)	72 (*p* = 0.00032)
Horizontal mean sway velocity (m/s)	0.278 (0.233–0.324)	0.187 (0.157–0.216)	69 (*p* = 0.00022)
AP sway radius (m/s^2^)	0.068 (0.061–0.075)	0.050 (0.043–0.057)	74 (*p* = 0.00042)
Horizontal sway radius (m/s^2^)	0.088 (0.077–0.098)	0.061 (0.055–0.067)	62 (*p* < 0.0001)
ML sway path length (m/s^2^)	5.23 (4.54–5.93)	3.22 (2.82–3.61)	38 (*p* < 0.0001)
Horizontal range of acceleration (m/s^2^)	0.541 (0.478–0.604)	0.382 (0.340–0.423)	63 (*p* = 0.0001)
ML range of acceleration (m/s^2^)	0.293 (0.247–0.340)	0.164 (0.147–0.182)	37 (*p* < 0.0001)
ML total spectral power (m^2^/s^4^)	0.812 (0.561–1.063)	0.230 (0.199–0.262)	27 (*p* < 0.0001)

*ML medial-lateral; *AP* anterior-posterior; units reflect measures are derived from an acceleration signal; significance defined as *p* < 0.00054 after Bonferroni correction

**Table 3:** Mean (95 % CI) measures of sway with significant group differences: eyes closed

*Instrumented measure of sway	Group with MS	Control group	Mann–Whitney U (*p*-value)
AP jerk (m^2^/s^5^)	0.274 (0.000–0.609)	0.027 (0.020–0.033)	60 (*p* < 0.0001)
Horizontal jerk (m^2^/s^5^)	5.72 (0.000–12.19)	0.521 (0.413–0.629)	57 (*p* < 0.0001)
ML jerk (m^2^/s^5^)	0.151 (0.000–0.335)	0.0066 (0.0051–0.0082)	33 (*p* < 0.0001)
Horizontal sway area (m^2^/s^4^)	0.146 (0.000–0.317)	0.012 (0.010–0.015)	47 (*p* < 0.0001)
95 % confidence circle area (m^2^/s^4^)	1.533 (0.317–2.749)	0.207 (0.167–0.247)	62 (*p* < 0.0001)
95 % confidence ellipse area (m^2^/s^4^)	1.043 (0.081–2.005)	0.128 (0.101–0.154)	43 (*p* < 0.0001)
AP root mean square (m/s^2^)	0.249 (0.175–0.323)	0.129 (0.118–0.140)	68 (*p* = 0.0002)
Horizontal root mean square (m/s^2^)	0.291 (0.195–0.388)	0.144 (0.131–0.156)	59 (*p* < 0.0001)
ML root mean square (m/s^2^)	0.140 (0.075–0.204)	0.057 (0.047–0.068)	58 (*p* < 0.0001)
Horizontal mean sway velocity (m/s)	0.689 (0.536–0.841)	0.403 (0.346–0.459)	76 (*p* = 0.00053)
AP sway radius (m/s^2^)	0.191 (0.139–0.242)	0.103 (0.094–0.113)	71 (*p* = 0.00028)
Horizontal sway radius (m/s^2^)	0.236 (0.166–0.307)	0.122 (0.111–0.133)	56 (*p* < 0.0001)
ML sway radius (m/s^2^)	0.101 (0.062–0.140)	0.046 (0.037–0.055)	57 (*p* < 0.0001)
AP sway path length (m/s^2^)	16.08 (11.68–20.48)	8.76 (7.82–9.69)	67 (*p* = 0.00018)
Horizontal sway path length (m/s^2^)	21.78 (15.52–28.04)	10.74 (9.68–11.81)	51 (*p* < 0.0001)
ML sway path length (m/s^2^)	11.28 (7.89–14.67)	4.54 (4.01–5.08)	30 (*p* < 0.0001)
AP range of acceleration (m/s^2^)	1.469 (0.928–2.011)	0.693 (0.636–0.751)	66 (*p* = 0.00016)
Horizontal range of acceleration (m/s^2^)	1.768 (1.020–2.516)	0.769 (0.705–0.834)	51 (*p* < 0.0001)
ML range of acceleration (m/s^2^)	0.928 (0.405–1.452)	0.307 (0.258–0.356)	48 (*p* < 0.0001)
AP total spectral power (m^2^/s^4^)	31.63 (7.17–56.10)	4.14 (3.42–4.85)	69 (*p* = 0.00023)
Horizontal total spectral power (m^2^/s^4^)	17.46 (4.57–30.34)	2.40 (2.02–2.78)	48 (*p* < 0.0001)
ML total spectral power (m^2^/s^4^)	12.92 (0.00–28.81)	0.641 (0.504–0.778)	31 (*p* < 0.0001)

*ML medial-lateral; *AP* anterior-posterior; units reflect measures are derived from an acceleration signal; significance defined as *p* < 0.00054 after Bonferroni correction

The stepwise logistic regression model identified sway path length and the range of sway acceleration amplitude in the medial-lateral direction and eyes-open condition as the only two significant independent predictors to differentiate subjects with MS from those without MS (model chi-squared = 34.55, *p* < 0.0001): specificity = 85 %, sensitivity = 90 %, accuracy = 87.5 %, area under receiver operating characteristics curve = 0.957 (0.90–1.0), positive likelihood ratio = 6 (2.09–17.21), negative likelihood ratio = 0.12 (0.03–0.44). Individually, the area under the curve for the range of acceleration amplitude alone was 0.908 (0.81–1.0), and that for sway path length alone was 0.905 (0.82–1.0). A cutoff value of 0.216 m/s^2^ for the range of acceleration amplitude provided 80 % sensitivity and 95 % specificity. A cutoff value of 4.0 m/s^2^ for the sway path length provided 85 % sensitivity and 80 % specificity.

### Correlating subject-reported measures with instrumented sway measures or clinical-exam measures

**Fig. 1:**
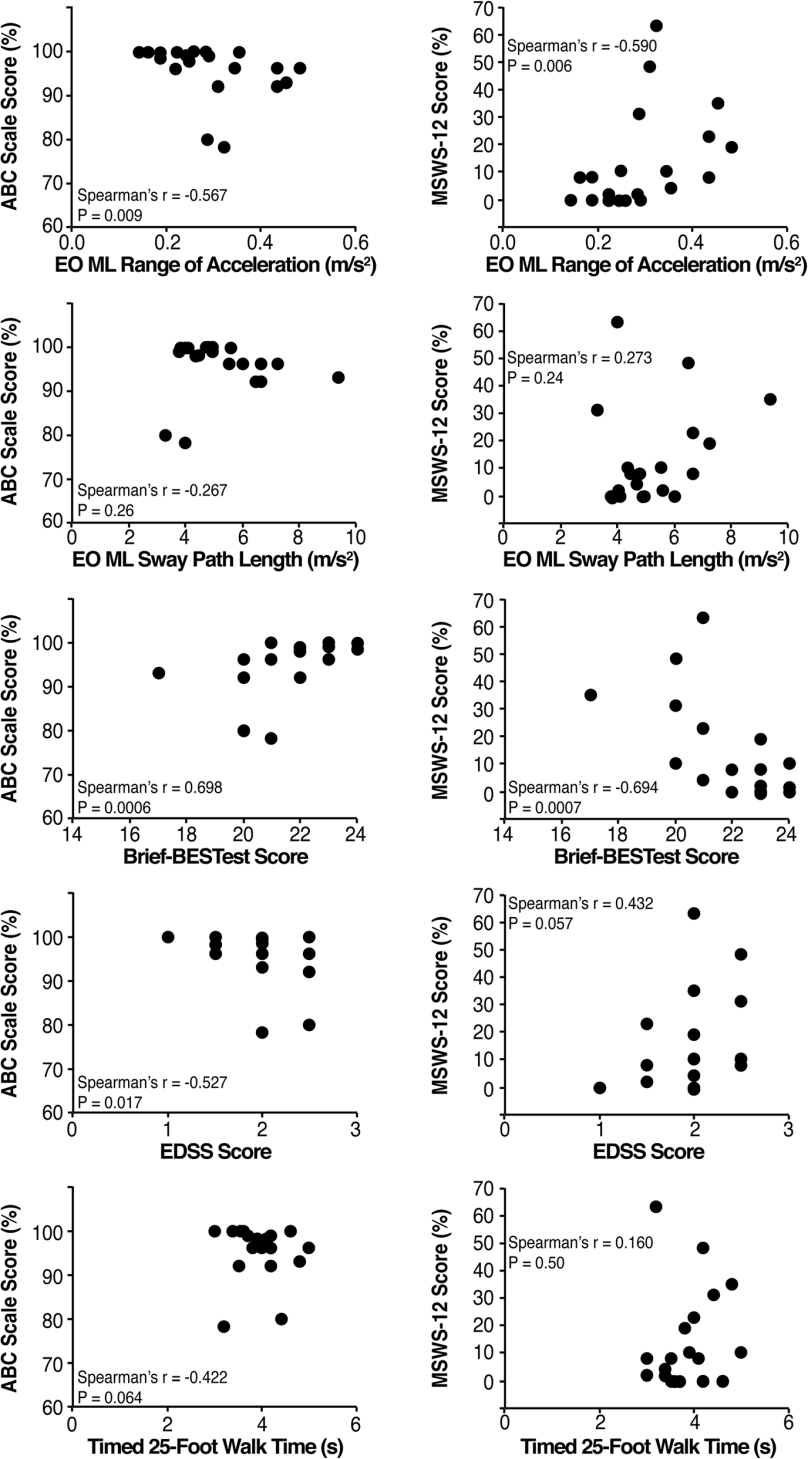
Scatter plots demonstrating the strength of Spearman’s correlations of subject-reported measures (ABC scale and MSWS-12) versus instrumented sway measures found to differentiate between people with and without MS (medial-lateral (ML) range of acceleration amplitude and sway path length in the eyes-open (EO) condition) as well as clinical measures of impairment and disability (Brief-BESTest, EDSS, and T25FW)

All correlation analyses with the subject-reported measures of balance and gait difficulty (ABC scale and MSWS-12) are illustrated in Fig. 1. As the significant independent predictors of group status, medial-lateral sway path length and range of sway acceleration amplitude were selected in the correlation analysis with subject-reported measures of balance and gait difficulty. The range of sway acceleration amplitude, but not sway path length, significantly correlated with ABC-scale and MSWS-12 scores. Although the T25FW did not generate significant correlations with ABC-scale or MSWS-12 scores, the Brief-BESTest significantly correlated with both self-report measures and the EDSS significantly correlated with ABC-scale scores but not MSWS-12 scores.

## Discussion

Our results demonstrate that instrumented measures of standing sway were able to differentiate subjects without MS from those with minimally disabling MS and clinically normal gait speed. Medial-lateral sway path length and range of sway acceleration in the eyes-open condition provided the strongest independent predictors of group status. The range of sway acceleration amplitude in the medial-lateral direction and eyes-open condition also significantly correlated with subject-reported measures of balance and gait difficulty. These subjects reported high levels of balance confidence and low impact of MS on mobility using these measures. These observations could reflect ceiling and floor effects of the ABC and MSWS-12, suggesting that these test instruments are not sensitive enough to detect initial changes in the quiet sway of a minimally impaired cohort. In contrast, gait speed (as measured by T25FW) was not associated with subject perceptions of balance and gait impairment; EDSS scores exhibited a weaker correlation with balance confidence and no significant association with self-reported gait impairment. The Brief-BESTest exhibited the strongest correlations to both the ABC-scale and MSWS-12 scores. Thus, the instrumented measure of range of sway acceleration amplitude in the medial-lateral direction appears capable of identifying balance impairment in minimally disabled patients with MS and with stronger associations with subject-reported experiences than those provided by the T25FW or EDSS exams.

Numerous studies have found balance impairment in individuals with MS at varying levels of disability [2]. Our MS cohort included individuals who were “minimally disabled” or “early-stage” as determined by EDSS scores and T25FW times that specify clinically meaningful benchmarks for disease progression and disability [8, 28, 29]. Although the definitions of “minimally” or “mildly” disabled have varied with methodology, our data also supports the identification of abnormalities of postural sway in people with MS at a similar level of disability in a number of instrumented studies [12, 22, 24, 25, 30–36].

Our results compliment past studies on postural sway in people with MS. In subjects minimally disabled with MS, past studies of posturography have identified higher sway velocities [25] and differences in the range of trunk roll [12] compared to control subjects without MS. In addition, center-of-pressure measurements in previous studies have demonstrated greater root-mean-square velocity [34] and altered medial-lateral and anterior-posterior sway variability with the removal of vision [30]. In our study, numerous measures in both the medial-lateral and anterior-posterior directions were also significant, and differences between subjects with and without MS were also evident in both the EO and EC conditions. Although many measures were identified as different between groups with the eyes closed, the stepwise regression analysis only included two measures derived from the EO condition. Some specificity may be lost for differentiating individuals with and without mild MS in the EC condition because the task is also challenging for healthy adults in the EC condition. As such, despite many impairments being evident with the eyes closed, the best predictors of early-stage MS were evident with the eyes open.

Sway velocity has also been reported to associate with clinical exams and self-report of balance impairment such as the Berg Balance Scale, ABC scale, and EDSS [1]. In contrast, some studies have demonstrated balance disorders undetectable by more widely used scales in people with MS [32, 33]. This study found that instrumented sway measures provided both the capacity to differentiate people with early-stage MS from people without MS and the capacity to correspond with subject-reported balance and gait difficulty, whereas the EDSS and T25FW either did not correlate as strongly with self-report measures or (by nature of our subject sample criteria) exhibited values that would not likely identify balance and mobility impairment in subjects with versus without early-stage MS.

This study’s objective was focused on the discriminative validity of instrumented sway measures. Thus, our control subjects without MS did not complete the clinical exam items. Unfortunately, we are therefore unable to compare the Brief-BESTest’s discriminative validity in this cohort of clinically minimal disability and normal gait speed. The mean and 95 % confidence interval of this study’s cohort with MS were relatively high, suggesting the Brief-BESTest would not be as powerful as the instrumented sway measures to discriminate people with versus without early-stage MS. Although speculative, if we examine each individual’s score compared to the 95 % confidence intervals of age-normative values presented in other studies, [9, 37] we identified nine of our 20 subjects with MS as having scores below these confidence intervals. Therefore, in combination with the correlation analysis that demonstrated significant correlations between Brief-BESTest scores and subject-reported balance and mobility difficulty, our results suggest at least a moderate discriminative validity for the Brief-BESTest. Compared to the sensitivity and specificity provided by the instrumented sway measures, though, we speculate that the Brief-BESTest’s accuracy would not likely reach similar levels. These combined results of the EDSS, T25FW and Brief-BESTest, therefore, suggest that people with mild MS can execute several motor tasks in the clinic without evident impairment based on visual analysis by a clinician, but subtle changes in balance are evident and people with mild MS are aware of the increased challenge to execute these tasks.

This study evaluated standing postural sway using inertial motion sensors while subjects stood on a compliant surface of medium-density foam under the supposition that the measurement system would be clinically feasible and the foam condition would better differentiate subjects with and without MS [23]. While numerous studies have demonstrated that posturography can differentiate balance control between subjects with and without MS [32], balance and mobility assessment using body-worn sensors and automated data processing software offer the potential of portability compared to traditional kinematic methodology as well as generalizability to multiple movement tasks while allowing clinically practical data collection [22]. In previous studies on minimally disabled people with MS, body worn gyroscopes identified significantly more trunk sway than in healthy control subjects [24] and significant differences in pitch angle range [36]. Body-worn sensors utilizing technology and algorithms similar to devices used in our study have also demonstrated significantly greater sway acceleration amplitude during quiet stance with eyes closed in subjects with MS compared to control subjects [22]. Unlike these prior studies, evaluation of stance on foam appears to provide strong levels of discriminative validity to identify people with minimally impaired MS from control subjects without MS. Body-worn sensors may offer the opportunity for sensitive, objective measures of assessment, yet provide clinical feasibility due to portability and automated algorithms for outcome processing. More data are needed to compare the benefits of using body-worn inertial sensors over other instrumentation methods.

## Conclusions

Postural sway abnormalities in subjects with MS who are minimally disabled were detected using wireless inertial sensors and may signify a superior sensitivity in the identification of balance impairment prior to the development of clinically evident disability. The relatively novel use of synchronized body-worn inertial sensors utilized in our study is the subject of increasing study in neurological disease [17, 21, 27, 38]. The improved ability to detect and analyze postural sway abnormalities through these methods may ultimately improve our understanding of the association between postural sway abnormalities and falls in patients with MS [39–41]. Development of longitudinal studies using synchronized wireless body-worn sensors, [26] particularly in minimally disabled cohorts, may provide important biomarkers for the early identification of individuals who may benefit from therapeutic interventions, as well as allow ongoing assessment of such disease modifying and symptomatic therapies to prevent falls before the accrual of significant disability.
